# Profiling the Mismatch Tolerance of Argonaute 2 through Deep Sequencing of Sliced Polymorphic Viral RNAs

**DOI:** 10.1016/j.omtn.2017.08.010

**Published:** 2017-08-24

**Authors:** Pantazis I. Theotokis, Louise Usher, Christopher K. Kortschak, Ed Schwalbe, Sterghios A. Moschos

**Affiliations:** 1 Department of Biomedical Sciences, Faculty of Science and Technology, University of Westminster, London W1W 6UW, UK; 2 Westminster Genomic Services, Faculty of Science and Technology, University of Westminster, London W1W 6UW, UK; 3 Department of Applied Sciences, Faculty of Health and Life Sciences, Northumbria University, Newcastle, Tyne and Wear NE1 8ST, UK

**Keywords:** siRNA, SNP susceptibility, RACE-SEQ, personalized medicine, oligonucleotides, RNAi, pharmacology, mechanism of action, shRNA, bioinformatics

## Abstract

Low allelic and clonal variability among endogenous RNAi targets has focused mismatch tolerance studies to RNAi-active guide strands. However, the inherent genomic instability of RNA viruses such as hepatitis C virus (HCV) gives rise to quasi-species mutants within discrete clones: this facilitates mismatch tolerance studies from a target perspective. We recently quantified the slicing imprecision of Argonaute 2 using small interfering RNA (siRNA) analogs of the DNA-directed RNAi drug TT-034 and next-generation sequencing of 5′ RNA ligase-mediated rapid amplification of cDNA ends (RACE-SEQ). Here, we present an open-source, customizable, and computationally light RACE-SEQ bioinformatic pipeline, describing adaptations that semiquantitatively report the impact of RNAi hybridization site mismatches from the target perspective. The analysis shows that Argonaute 2 has a substitution-specific, 3- to 5-log activity window between fully complementary targets and targets with mismatches across positions 10–11. It further focuses the endonucleotic Slicer imprecision around positions 13–17, demonstrating its dependence on guide strand central region complementarity, and potentiation by even a single mismatch. We further propose pharmacogenomics value in testing endogenous targets using recombinant replicon systems and RACE-SEQ to report the pharmacodynamics of sequence-specific oligonucleotide therapeutics against all possible polymorphisms in a population, in a minimally biased, patient-free manner.

## Graphical Abstract

**Figure:**
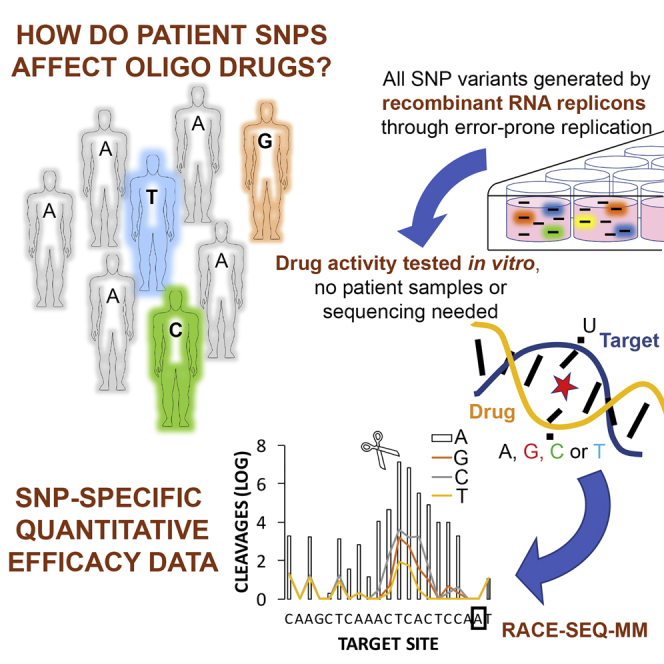

## Introduction

RNAi is the biological process by which endogenous or exogenous RNA molecules drive sequence-specific changes in gene expression through RNA-induced silencing complexes. Study of the intrinsic pathways and co-factors involved in RNAi has shed light on some of the mechanics of gene expression regulation, spurring significant progress in the development of associated technologies.^1^ Thus, since its unexpected discovery,^2^ RNAi has matured into numerous applications in plants and is now poised to achieve regulatory approval for clinical use. Key to clinical success, however, has proven to be the provision of sustained and pharmacologically rational evidence of an on-target mechanism of action across the drug development pipeline.^3^

At the core of RNAi, the RNA-induced silencing complex (RISC) involves an Argonaute protein loaded with a single, ∼21-nt-long RNA strand, commonly referred to as the active or guide strand.^4^, ^5^ The best-understood mechanism that RISC uses to recognize RNA targets appears to be the so-called guide strand “seed” sequence between bases 2–8 from the 5′ end of the guide strand,^6^ short enough to implicate potentially thousands of transcripts as putative targets per guide strand.^7^ Thus, target recognition following RISC binding sequesters and represses RNA translation. In mammals, only Argonaute 2 (AGO2) possesses the so-called “Slicer” capacity to cleave its targets,^8^, ^9^ an endonuclease function that generates a novel, uncapped 5′ end on RNA, preventing transcript translation and enabling target RNA degradation.^10^ Importantly, slicing is principally focused opposite positions 10–11 from the 5′ end of the guide strand^11^, ^12^ and seems to require full Watson-Crick base pairing across these bases.^13^ Thus, detecting a novel 5′ end on an RNA target at this location provides explicit evidence of an on-target mechanism of action for Slicer RNAi. Together with the seed sequence, these two features have preoccupied mechanistic studies of novel RNAi therapeutics.^14^

Historically, the low-resolution 5′ RNA ligase-mediated rapid amplification of cDNA ends (5′ RLM-RACE)^15^ method has been used to provide qualitative evidence of the mechanism of action of Slicer RNAi.^16^, ^17^ More recently, medium- and high-resolution quantitative methods, such as next-generation sequencing of total RNA (RNA-SEQ) and 5′ RLM-RACE products (RACE-SEQ),^18^, ^19^ respectively, have been developed. Importantly, the high resolution of RACE-SEQ has confirmed that slicing of a given target can be considerably less precise than originally thought. Thus, up to a quarter of all Slicer cleavage products generated by a single guide strand might occur even as many as +5 bases further away from the primary cleavage site, toward the 5′ end of the RNA target molecule.^18^ Although slicing imprecision was previously observed after Sanger sequencing of 5′ RLM-RACE products,^20^, ^21^ research has focused on minimizing the off-target effects of RISC in terms of unintended transcript engagement (e.g., due to seed-mediated target recognition).^22^ The impact of target variability beyond single nucleotide polymorphisms (SNPs),^23^ heterozygocity,^24^ or mutational escape in RNAi-targeted viruses^25^ remains unexplored.

The original description of RACE-SEQ involved synthetic, short interfering RNA (siRNA) mediators of RNAi targeting hepatitis C virus (HCV), as modeled in a well-established, in vitro HCV RNA replicon system.^18^ HCV is a positive RNA strand virus that encodes an RNA-dependent RNA polymerase (RdRP) required for the replication of the viral genome.^26^, ^27^ This enzyme, which is also used for replicon maintenance in vitro, lacks any proofreading function,^28^, ^29^ making HCV replication highly error prone.^30^, ^31^ As a result, it is common to detect populations of HCV genome sequences within single viral genotype infections, even when using Sanger sequencing; these are referred to as HCV quasi-species.^32^ However, unlike the restricted sequence variability of host transcripts because of low somatic mutation rates,^33^ the inherent genomic variability of HCV presents a unique opportunity to study how mismatches arising randomly on targets may affect the specificity of Slicer.

**Figure 1:**
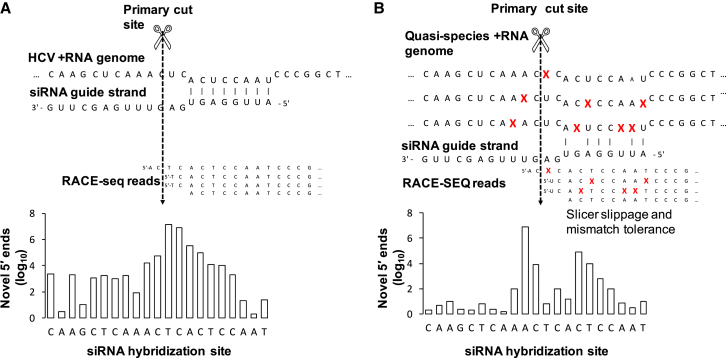
Using RNA Replicon Systems and RACE-SEQ to Query and Enumerate the Susceptibility of Target Mismatches to Defined RNAi Mediators in a Patient-free, Agnostic, and Minimally Biased Manner (A) Slicing of a given RNA target (e.g., the RNA genome of HCV) generates novel 5′ ends at a primary cut site, principally opposite positions 10–11 from the 5′ end of guide strand loaded onto an AGO2-RISC. (B) The 5′ RACE-SEQ assay uses a gene-specific primer positioned downstream of the cut site on a target RNA to digitally report both the primary and additional fragments generated (e.g., degradation products starting to the right of the cleavage site or other guide strand-induced fragments starting beyond the Slicer primary active site). Due to HCV’s high mutation rates, various polymorphisms (indicated by “X”) result in multiple quasi-species of virus arising within the context of a single viral genotype. We thus explore how random and specific mismatches in a given target affect RACE-SEQ outputs, to map how target variability affects Slicer susceptibility semiquantitatively and in a base-by-base manner.

Thus, in their original study, Denise et al.^18^ used unmodified siRNA analogs of the principally active guide strands processed out of the first-in-class biotherapeutic RNAi drug TT-034. This proposed single-shot treatment for HCV is an adeno-associated virus 8 gene therapy that targets hepatocytes to express three short hairpin RNA (shRNA) mediators of RNAi designed to cleave the HCV RNA genome.^34^ The authors used sub-maximal (30 nM) concentrations of these three siRNAs, independently reverse transfected into Huh7.5 cells harboring a HCV replicon. At 48 hr, RNA was harvested, subjected to 5′ RLM-RACE using a shortened 5′ RNA adaptor, strand-specific gene-specific primers, and touch-down PCR, to yield ∼85-base pair (bp) amplicons. The amplicons were then subjected to Illumina deep sequencing in a paired-end fashion; due to their short size, these amplicons were thus subjected to high-accuracy duplex sequencing.^35^ Novel 5′ ends were enumerated after adaptor trimming and alignment to the reference replicon genome using a proprietary bioinformatic pipeline. We postulated that stringent data mining of such RACE-SEQ datasets could yield additional valuable information: the highly variable nature of the HCV genome should enable querying of Slicer precision against HCV quasi-species targets with one or more mutations natively found in these datasets. In addition, by carefully controlling mismatch tolerance during SEQ data alignment, we should be able to examine cleavage activity on the HCV quasi-species in a base-by-base manner (Figure 1).

**Figure 2:**
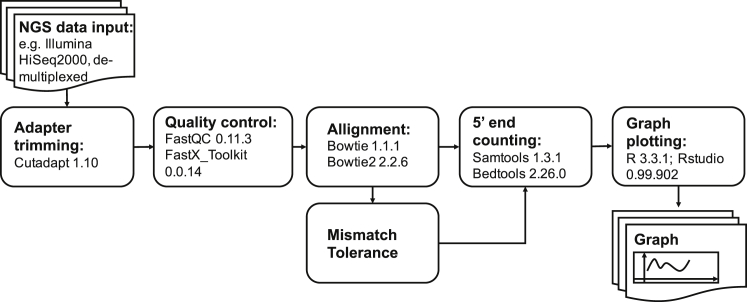
The Open-Access Bioinformatics Pipeline for Low Computational Overhead RACE-SEQ Analysis, Including Exploration of Mismatch Tolerance and Mismatched Target Recognition by Slicer RNAi

Here, we therefore expand upon the siRNA RACE-SEQ data generated by Denise et al.^18^ to explore the susceptibility of the HCV replicon quasi-species to the three guide strands principally produced by TT-034. We demonstrate how the specificity and relative potency of RNAi mediators against all possible variants of a target sequence can be determined agnostically and in a minimally biased manner, at the single nucleotide level, simply by using replicon systems. Moreover, we present a low computational overhead pipeline freely available online under the GNU General Public License for the processing of RACE-SEQ data (Figure 2), and we provide simple pipeline adjustments for evaluating the tolerances of an RNAi therapeutic to target sequence variation.

## Results

### Low Computational Overhead Data Processing

**Figure 3:**
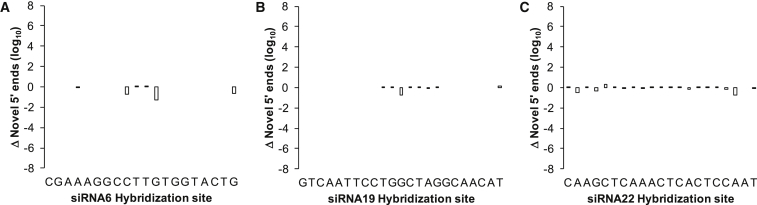
Effect of RACE-SEQ Data Processing Using a Proprietary High Computational Overhead Pipeline versus the Newer Pipeline Presented Herein for Three Separate siRNAs (A–C) siRNA6 (A), siRNA19 (B), and siRNA22 (C) target independent sites on the HCV replicon genome. Driving Slicer-mediated cleavage of the HCV replicon RNA genome suggests less than 0.001% variance to maximal incidence counts. Changes in novel 5′ read counts (y axis) at each position on the HCV genome targeted by an siRNA (x axis; HCV genome in a 5′ to 3′ fashion) is represented in log10 scale reflecting the data distribution range for 5′ end incidence counts.

The high processing speed and memory efficiency of the Cutadapt package is ideal for implementation in RACE-SEQ analyses due to its ability to define RACE adaptor location (5′ or 3′), thereby enabling more precise trimming. This means our pipeline is also easily implementable in 3′ RACE-SEQ studies (e.g., to understand cleaved RNA degradation in a 3′→5′ manner). To maintain 100% complementarity with the adaptor and consistency with Denise et al.,^18^ Cutadapt was set to “-e0 --no-indels --discard-untrimmed.” Thus, all accepted reads would contain the exact 5′ RACE adaptor sequence used experimentally and no other RNA ligation products (Table S1). In addition, FastQC confirmed that all the adaptor-trimmed reads had quality scores well within the normal distribution and sequence lengths of between 45 and 50 bps trimmed off a ∼85-bp read containing a 30-nt RNA adaptor (Figure S1). Furthermore, collapsing the datasets with fastx_collapser into files enumerating read incidence also significantly reduced data complexity. This illuminated the variety and prevalence of the Slicer products generated. Thus, only 1,644 different sequences made up for the 2.5 million reads that aligned to the HCV replicon genome (GenBank: AJ238799) using Bowtie1, representing 78.15% of the dataset. In stark contrast, 298,000 different sequences corresponded to apparently non-HCV data (Table 1). This meant that the ∼2-log reduction of reads due to collapsing could be extended by another ∼2 logs simply by removing the highly diverse read dataset that did not align to HCV, ultimately reducing the 12.5-GB initial dataset files to 18.5 MB. Crucially, despite the drastic reduction in file size, enumeration of novel 5′ ends did not introduce any appreciable artifacts relative to the scale of the novel 5′ end count dataset (Figure 3).

**Table 1:** Effect of Read Collapsing on Read Count and Reference Alignment with No Mismatches Tolerated

Reads	Before Collapsing		After Collapsing	
	Absolute	Relative (%)	Absolute	Relative (%)
Aligned to HCV	20,505,001	78.15	1,644	0.55
Unaligned	5,733,712	21.85	297,932	99.45
Total	26,238,713	100.00	299,576	100.00

On the other hand, given the significant size (21.9%) of apparently unaligned reads within the dataset (5.7M; Table 1), the unaligned data fraction was collapsed and compared to the HCV replicon genome by multiple sequence alignment. This analysis revealed that 28.1% of the unaligned dataset contained sequences with minimal variation (e.g., mismatches, indels) to the HCV reference genome (Figure S1). Given the error rate of HCV’s RdRP and the relative size of this HCV-related read population, this data subset was interpreted as a sequencing signature of the HCV quasi-species within the 5′ RACE-SEQ data. Thus, re-aligning the reads against the HCV replicon, this time accepting up to 3 mismatches to the replicon reference sequence, indicated that up to an additional 1.6M reads might indeed arise from naturally occurring HCV replicon variants.

### Replicon Quasi-species Are Also Cleaved by Slicer

We next proceeded to examine in detail the impact of tolerating mismatches to the 5′ RACE-SEQ profile. To begin with, we aligned the 5′ RACE-SEQ reads to the HCV replicon reference genome, configuring Bowtie1 to tolerate an increasing number of random mismatches; the results were then plotted as the increase in novel 5′ end counts for each additional mismatch tolerated (Figure S2). This indicated that the overall cleavage profiles were not affected for siRNA19 and siRNA22. Thus, with position 10–11 remaining the primary cleavage point, the relative proportions of novel 5′ ends among mismatched reads generally followed the same trends as those of fully aligned reads, whether one, two, or three mismatches were tolerated.

On the other hand, with siRNA6, substantially more novel 5′ end counts were observed downstream of the cleavage site: curiously, in some cases, the mismatch-containing reads were well in excess of counts obtained in the absence of mismatches, making the total number of siRNA6 cleavage events 234× higher than that observed for siRNA19. At first glance, these results raised the prospect of extensive quasi-species sequence variability within the siRNA6 hybridization site, against which siRNA6 might be more active. However, we considered this highly unlikely for three reasons. First, the siRNA6 target site lies within the IIId domain of the 5′ non-coding region of HCV, a highly structured RNA conserved across HCV genotypes^36^ and even into picornaviruses.^37^ Second, historical in vitro^38^ and in vivo^39^ data with a semiquantitative RT-PCR assay indicated an overall siRNA6 activity level lower than that of siRNA19. Third, reads starting downstream of an siRNA primary cleavage point, shorter by ∼10 nt, would be expected to exhibit an increased likelihood of type I error (false positive alignments) with increasing mismatch tolerance.

**Figure 4:**
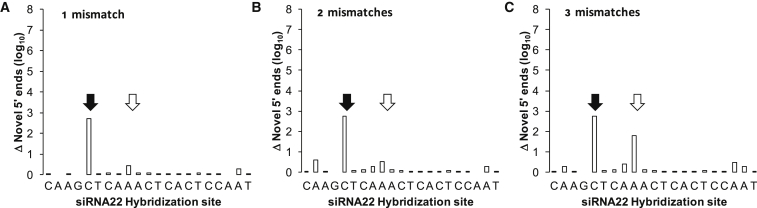
Impact of Increasing Random Mismatch Tolerance in siRNA22 RACE-SEQ Reads upon Bowtie1 Alignment to the HCV Genome (A–C) The effect of 1 (A), 2 (B), or 3 (C) random mismatches in read alignment is expressed as the increase in novel 5′ ends (y axis) generated at a given position of the HCV genome (x axis), revealing position 13 (white arrow), but not position 17 (black arrow), variability to be dependent on mismatch tolerance extent.

Furthermore, looking at the log-scale differences in total novel 5′ end counts between the three siRNAs (10^3^ for siRNA6 versus 10^7^ for siRNA22), we considered the value of normalizing the mismatched alignment counts for the amount of data available per position. To do this, we plotted the cumulative log-scale differences in novel 5′ end counts at increasing levels of mismatch tolerance (Figure S2). This analysis highlighted how the novel 5′ end count increases across the shorter G1–G6 reads of the siRNA6 hybridization site increased alongside the extent of mismatching permitted. No substantial (>10×) differences were observed across the siRNA6 central or upstream region, nor indeed across the entirety of the siRNA19 hybridization site. Interestingly, in the largest dataset by read depth (siRNA22; Figure 4), a nearly 1,000× increase was observed in novel 5′ ends at position C17 from the 5′ end of siRNA22. In contrast to C17, increases at position A13 seemed to reflect the extent of mismatch tolerance permitted. Taken together, these results reaffirmed the value of considering 5′ RACE-SEQ data in log scale (i.e., similar to RNA-SEQ). Furthermore, they indicated that mining replicon quasi-species susceptibility through mismatch tolerance would be more robust for longer 5′ RACE-SEQ reads. Thus, as little as a single mismatch could be shown to potentiate cleavages beyond the primary Slicer site, such as at position C17 in the siRNA22 hybridization site. However, as this effort did not shed light on where these mismatches might be located, we decided to explore how each possible base substitution in the siRNA22 hybridization site affected the ensuing novel 5′ end profile.

### Replicon Quasi-species Analysis Distinguishes Polymorphic Targets Subject to RNAi-Directed Cleavage

**Figure 5:**
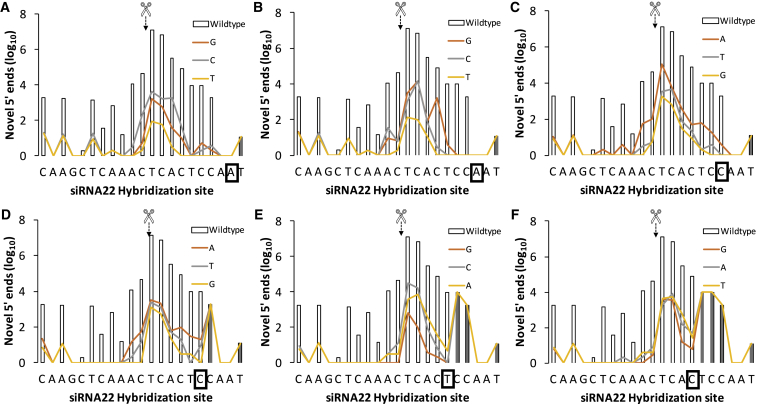
Effect of Specific Nucleotide Substitutions across from the Seed Region of siRNA22 on Novel 5′ End Generation in the Genome of a HCV Replicon (A–F) The specific substitution within the seed region [position 2 (A) to position 7 (F) in a 5′ to 3′ direction for siRNA22; i.e., right to left] is highlighted by the boxed nucleotide on the x axis of each panel. Scissors highlight the Slicer primary active site. Bar plots depict the cleavage profile generated in the wild-type Con1B HCV replicon reference sequence. A differentially colored line represents the effect of each possible base substitution within the boxed base. Reads starting at positions downstream (to the right) of the iteratively substituted nucleotide (box) are not affected by substitutions and therefore have no effect on novel 5′ end counts (shaded barplots), resulting in overlapping substitution effect lines.

**Figure 6:**
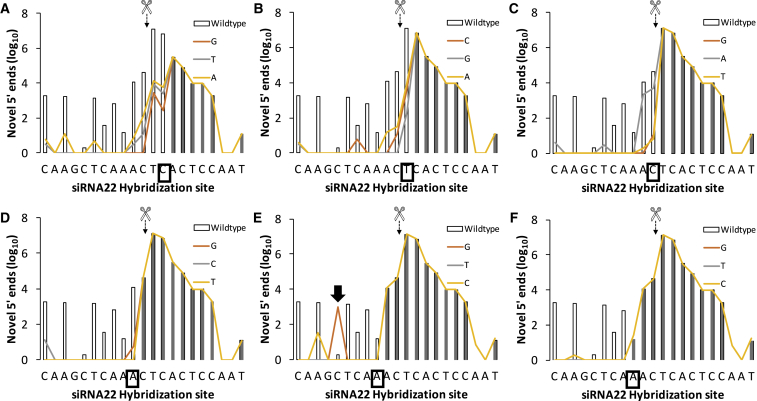
Effect of Specific Nucleotide Substitutions across from the Slicer Active Site for siRNA22 on Novel 5′ End Generation in the Genome of a HCV Replicon (A–F) A boxed nucleotide on the x axis highlights the base substituted within each panel across the Slicer active site (scissors) between position 9 (A) and position 14 (F) in a 5′ to 3′ direction of siRNA22 (i.e., right to left). Bar plots depict the cleavage profile generated in the wild-type Con1B HCV replicon reference sequence. A differentially colored line represents the effect of each possible base substitution within the boxed base. Reads starting at positions downstream (to the right) of the iteratively substituted nucleotide (box) are not affected by substitutions and therefore have no effect on novel 5′ end counts (shaded barplots), resulting in overlapping substitution effect lines. The source of the C17 novel 5′ end count increases identified in Figure 4A is highlighted by the black arrow.

To achieve this using Bowtie1, we used an R script and carried out iterative alignments with *-v* set to 0 against a set of HCV replicon reference genome variants manually derived from GenBank: AJ238799. These were produced to cover all possible SNPs within the siRNA hybridization site seed sequence (Figure 5) and the central, Slicer active site-spanning region (Figure 6). Thus, each of these 36 alternative reference genomes would feature one of three possible substitutions at a specific position within the siRNA binding site (e.g., in siRNA22, 10T to 10A, or 10C or 10G; Figure 6B), and novel 5′ ends would be accepted only when reads were fully complementary to the specific SNP-containing genome. To allow direct comparisons of the effect of each polymorphism on novel 5′ end profiles, we overlaid the three alternative profiles as color-coded line graphs against the wild-type histogram profile, highlighting the substitution position on the x axis.

As expected, novel 5′ read counts downstream of the base substitution were not affected (Figures 5 and 6; gray bars), as these reads did not include the substituted base. However, remarkable and variable differences in novel 5′ end counts were observed upstream (i.e., when incorporating base substitutions in read alignment). Crucially, substitutions at position 10 reduced Slicer activity by 3 (10T→C or A) or 4 logs (10T→G) but did not eliminate it. Moreover, the 3-log increase in read counts at C17 observed under random mismatch analysis was identified as an A13→G polymorphism.

## Discussion

Since its inception, RACE-SEQ has been used by others^17^, ^19^, ^40^ to enhance evidence of RISC-mediated target cleavage precision in preparation for pre-clinical and clinical studies. Two rate-limiting factors in the wider adoption of the method have been cost, associated with sequencing depth requirements, and the complexity of data processing. As we progress the method to medium-throughput deep sequencer compatibility (e.g., Illumina MiSeq and the Ion Torrent Personal Genome Machine) for reliable routine use, here we present a simple and computationally lean approach to RACE-SEQ data handling and representation. The resulting “RACE-SEQ lite” pipeline reduces raw data size by ∼4 logs and enables wider technique adoption by the community. This is illustrated by the computational analysis of the data presented in this article, which was performed on a dual core, 4-GB RAM laptop with a 250-GB hard drive: simple RACE-SEQ analysis to 5′ incidence graph production took approximately 20 min from a 12.5-GB .fastq file, while the 36 reference genome alignment and mismatch tolerance analyses took 9.6 hr to process. In developing this “RACE-SEQ lite” pipeline, we also took the opportunity to explore how sequence variability in RISC targets, as opposed to guide strand engineering, drives the mismatch tolerance of siRNAs. In this sense, data from RNAi applied on a HCV-based RNA replicon system were ideal: the well-documented error-prone replication of HCV naturally gives rise to sequence variance within the intended RNAi target. One could therefore explore the effects of this variance simply by actively looking for mismatches within the RACE-SEQ data. Such an extent of sequence variability is not normally easy to study with transcripts encoded in a DNA genome. However, this kind of analysis is useful because it permits the preemptive minimally-biased study of the impact of any possible polymorphism to the efficacy of RNAi treatment.

We therefore initially explored how tolerating random mismatches during read alignment would influence the incidence of novel 5′ ends generated on siRNA target regions. We reasoned that sequencing error would result in increases randomly distributed across the entire siRNA target site and would thus have no effect on overall cleavage profiles. In addition, smaller RNA fragments terminating proximally to the guide strand 5′ end would be also expected to randomly align to the reference genome as the number of mismatches tolerated was increased. While this indeed was the case for siRNA6 and siRNA19, analysis revealed positions 13 and 17 from the 5′ end of the siRNA22 guide strand as interesting exceptions (Figure 4). Crucially, the number of novel 5′ ends increased by ∼3 logs at position 17 irrespective of the number of mismatches tolerated. In contrast, the number of 5′ ends starting at position 13 increased in line with the extent of mismatch tolerance. This difference indicated that the observation at position 17 was unlikely to be an artifact of aligner tuning, but an indicator of an alternative cleavage site at this position, found exclusively among replicon sequence variants with one or more mismatches to siRNA22. Curiously, these new read counts at position 17 were comparable to observations across positions 14–17 in the absence of mismatches (Figure 5). Thus, it appears that the activity of siRNA22 in this area might be consistent whether targets have none, 1, 2, or 3 mismatches to the siRNA, and that this activity is ∼3–4 logs lower than that normally observed at position 10–11.

Importantly, this evidence builds upon the observation of Denise et al.^18^ that up to 25% of all novel 5′ ends produced by siRNA19 occurred across positions 11–15, a range that partially overlaps with positions 14–17 in the siRNA22 target site. It is therefore not surprising that mismatch tolerance analysis of the siRNA19 RACE-SEQ dataset revealed no new or substantially enhanced cleavage points across positions 14–17 (Figure S3). However, together with the siRNA22 mismatched read data, it would appear that cleavage within 14–17 can vary by as much as 4 logs. Whether a secondary cleavage site also exists in siRNA6 in this region is presently unknown, probably because of the low, 3-log dynamic range in this dataset on account of lower sequencing depth. Indeed, all of the novel 5′ ends in the siRNA6 target site revealed through mismatch analysis depended on the extent of mismatching permitted (Figure S4). Moreover, pipeline comparisons (Figure 3) suggested the siRNA6 dataset to be less resilient, a common indicator of inadequate sequencing depth. On this basis, we would argue that at least 10M 5′ RACE-SEQ reads are required per RISC target site to determine reliably both on- and off-target Slicer activity. Importantly, this is consistent with evidence from systematic studies on minor variant detection for HCV using next-generation sequencing.^41^

Nevertheless, the data for siRNA19 and siRNA22 implicate positions 14–17 as the putative focal point for a potentially secondary slicing site, which can be potentiated by as little as a single mismatch. Notably, such observations have also been reported by others, beyond HCV replicon systems, in vivo, against endogenous targets. Thus, Ganesh et al.^19^ reported evidence of <5% incidence of novel 5′ ends at position 14 for at least two of the five mice treated with a DICER-substrate siRNA. Whether lower-level activity was also present among the remaining animals remains unclear due to percentile data scaling in the report by Ganesh et al. and lack of clarity on coverage of the slicing site. Interestingly, this putative secondary Slicer active site spanning positions 14–17 of the guide strand is in accordance with the transitional nucleation model^42^ that favors a 3′ end guide strand interaction around positions 13–16 through restricted seed sequence engagement. More importantly, it permits for guide strand-to-target interaction slippage, which could account for observations of imprecise Slicer-mediated action upstream of position 10–11. Put simply, the 3′ end guide strand interaction model could account for Slicer-mediated cleavage induction across positions 13–16 with lower affinity to the canonical model, or RISC loading driven by guide strand slippage and not by Slicer imprecision. It is unclear at present, however, to what extent the stability of the RNA cleavage products arising at this position confounds the pinpointing of a putative secondary Slicer active site suggested through RACE-SEQ. In other words, the primary novel 5′ end peak in this region may shift to the right simply because of exonuclease activity.

A simpler interpretation of our observations would be evidence of RNA destabilization achieved by non-Slicer Argonaute RISC activity. However, this is incompatible with the 3′ end de-adenylation or 5′ end de-capping and 5′→3′ degradation mechanism proposed for endogenous transcripts.^42^ HCV and its replicon systems lack a 5′ cap^43^ or a poly(A) tail and rely on RISC complexes to promote their stability.^44^ Alternatively, RISC recruitment to sites other than the microRNA-122 recognition elements on the HCV genome^44^ could disturb the protective mechanism of microRNA-122 either directly or via Argonaute sequestration, thereby permitting replicon degradation by the RISC-associating exonucleases XRN-1 and XRN-2,^45^, ^46^ in addition to Slicer activity on the siRNA target site. The present data, however, do not support this hypothesis: treatment with control siRNAs would have resulted in de-protection of the replicon and RACE-SEQ-mediated detection of these secondary sites but not the Slicer site at position 10–11. Instead, we detected cleavages with the siRNA target sites in the control samples used by Denise et al.^18^ only when we accepted one or more mismatches during read alignment. Importantly, these occurred randomly across the target site and increased alongside the number of mismatches tolerated, suggesting nothing more than sequencing or mismatched alignment artifacts.

With random mismatch tolerance evidencing what we interpreted as additional RNAi activity onto the HCV replicon quasi-species, we next set out to map the effects of specific mismatches on Slicer function as reported by RACE-SEQ, specifically in siRNA22. To do this, we plotted the effects of all possible nucleotide substitutions across the seed region (Figure 5), and the primary Slicer cleavage site (Figure 6), in terms of novel 5′ incidence on the siRNA22 target site, as reported by the same RACE-SEQ biological dataset produced by Denise et al.^18^ Interestingly, forcing specific mutations in the reference sequence resulted in as much as a ∼4-log decrease in the number of novel 5′ ends (e.g., at position 10–11). This was consistent with the changes enumerated by accepting random single mismatches on novel 5′ end counts (Figure 4). Closer examination indicated that mismatches within the seed region exerted a substantial effect on siRNA Slicer activity, but did not eliminate it (Figure 5): cleavage at position 10–11 remained the primary activity of Slicer RISC as enumerated by RACE-SEQ. Nevertheless, the substantial silencing potential demonstrated through this analysis, even when >1 polymorphism/mutation arises within a guide strand hybridization site, may explain why resistance evolution studies with RNA viruses often report stabilizing mutations beyond the RISC binding site.

Looking at the relative impact of different mismatches, the effect of specific base substitutions within a defined position was either negligible, or no more than 1.5 logs, and was more pronounced for positions 2–4 in the seed region (Figures 5A–5C). Here, A→T transversions at positions 2 and 3 and C→G at position 4 had the strongest effect, indicating that M→K transversions at these locations may reduce cleavage at the primary active site of Slicer. The effect of mismatches at position 5 appeared less sensitive to specific types of substitutions (Figure 5D), expanding on previous observations regarding the effect of a G:U wobble in the context of otherwise full complementarity between miRNA-196 and HOXB8.^47^ However, we noted that a C→G transversion at this position eliminated novel 5′ generation at position 5, with a C→K transversion affecting novel 5′ end generation at positions 6 and 7 too. Similarly, A→Y transversions at position 3 eliminated novel 5′ end detection at position 7, whereas A→G transition only reduced counts by ∼1 log. These observations point away from cytosolic adenosine deaminase activity^48^ or exonuclease activity on fragments generated by cleavage at position 10–11 and in the direction of transversions in overcoming RNAi-induced target restriction. Thus, given the known preference of RNase A for YpR and Y_n_ stretches,^49^ one would expect changes that would increase RNase A susceptibility across the Y-rich 3-7 region to consistently reduce the incidence of novel 5′ ends. However, this is prominently not the case, as 4C→G and 5C→G also drop read counts. These results may therefore indicate Slicer slippage toward the 5′ end of the guide strand in addition to 3′ end slippage proposed by Denise et al.,^18^ and they suggest that off-target transcripts with seed sequence transversions might be less susceptible to RISC recognition. Alternatively, cytosolic RNase activity engaged in Slicer product decay (XRN enzymes^45^, ^46^) might be responsible for these observations. However, as the substrate preferences of these enzymes are not as well understood, and the shorter 5′ RACE-SEQ reads generated in this region might be misleading as observed for siRNA6, additional scrutiny is necessary before making conclusive statements over the slippage reach of Slicer or off-target transcript prediction.

Nevertheless, it is noteworthy that our observations are in agreement with a recent report by Geller et al.^50^ on the role transversions may play in HCV drug resistance evolution. Thus, both reports highlight an overrepresentation of A→G and U→C transitions in HCV replicons. Crucially, like Denise et al.,^18^ Geller et al.^50^ used bi-directional sequencing (duplex sequencing)^35^ to reduce the Illumina per-base read error rate in their studies to 8.4 × 10^−8^. Thus, Denise et al.^18^ adopted pair end sequencing over ∼85-bp RACE amplicons with no interspersed sequences between the paired reads, effectively applying what was later coined as duplex sequencing^35^ to the 5′ RLM-RACE amplicons. Interestingly, this error rate also matches the number of relevant RNA templates available to 5′ RACE-SEQ enumeration. Accordingly, the data discussed herein were produced from 96-well experiments seeded with 20,000 cells, each containing ∼40,000 copies of the Con1B replicon RNA (>8 × 10^8^ RNA templates total), the sum of which was subjected to 5′ RACE-SEQ. On this basis, we believe a 10-read (1 log_10_) threshold is justifiable across the 8-log_10_ read dynamic range of the Denise et al.^18^ dataset. Importantly, Geller et al.^50^ also evaluated the per-base mutation frequency for HCV replicons: the target site mutation frequency of siRNA22 (Figure S7) averaged 1.97 × 10^−4^ ± 9.72 × 10^−5^ in their study and reported minimal variance between replicon lines. This mutation frequency is consistent with our hypothesis that the assay data comfortably sample quasi-species sequences and are comparable to the novel 5′ end counts obtained (e.g., at mismatched 10T for siRNA22). It also suggests that detecting dual or triple mutant variants (2–3 mismatches) will be difficult, thereby constraining quasi-species analysis to reads containing only a single mismatch. Moreover, the 1.97 × 10^−4^ mutation frequency defined by Geller et al.^50^ is practically identical to the previously empirically derived, 20,000× coverage recommendation for minor variant detection in HCV-infected patients.^41^ It follows therefore that per-base coverage would need to substantially exceed 2 × 10^4^ to allow for reliable and quantitative detection of quasi-species variants within 5′ RACE-SEQ data. Indeed, coverage across the target site of siRNA22 spans 2.70 × 10^4^- to 2.22 × 10^7^-fold (Figure S7), with the 5′ end sensitivity of the RACE-SEQ assay and the dominance of Slicer fragments starting at position 10–11 explaining the ∼3-log_10_ coverage range. Most notably, where RACE-SEQ data coverage exceeds the per-base mutation frequency identified by Geller et al.^50^ threshold by more than 3 orders of magnitude, novel 5′ ends in quasi-species variants involving a specific single base substitution are comfortably detectable, even at coverages approaching 10,000× (e.g., Figure 5C). Taken together, these parameters strongly suggest that our observations regarding specific base substitutions are not the result of sequencing errors, but a true representation of the combined relative incidence of replicon quasi-species with a single base substitution within the specific siRNA target sites, and their individual susceptibility to Slicer cleavage.

Beyond the seed region and primary cleavage point, mismatches across positions 2–3 were not restrictive upon the secondary cleavage point(s) detected closer to the 3′ end of the hybridization site of the siRNA (positions 11 and beyond). However, we observed the opposite effect as we began forcing mismatches closer to the primary active site of Slicer (position 5 onward; Figures 5D–5F). This culminated with substitutions at positions 10–12 (Figure 6), suggesting that hybridization across the center of the guide strand and target is essential for cleavages to occur across positions 14–17, whatever the underlying mechanism(s) might be. The most unexpected finding, however, was that substitutions at position 10 did not eliminate novel 5′ end generation, but reduced it only by 3–5 logs (Figure 6B). This finding has important implications on the relative activity of allele-specific RNAi and, indeed, other similar oligonucleotide therapeutics. Thus, allele-specific RNAi^51^ involves guide strands designed against deleterious alleles in heterozygous cells wherein imprinting is not involved in selective allele expression and pivots on differential complementarity across the primary slicing site between the two alleles. As a result, mutant transcripts are sliced, permitting wild-type alleles to restore a healthy phenotype. Our evidence of cleavage even on transcripts mismatched at positions 10 and 11, however, suggests that the selectivity of allele-specific RNAi will more likely depend on classical enzyme kinetics: substrate concentration and affinity (RISC substrate on-rates, slicer cleavage rates, and RISC product off-rates). Thus, slicing of the healthy, mismatched allele transcript will occur and increase when its concentration exceeds the substrate dominance of the mutant transcript (e.g., due to epigenetic expression regulation or homeostatic factors relating to cellular phenotype, feedback loops, etc.). Consequently, the kinetics of Slicer selectivity against mismatched targets become increasingly less relevant in an allele-specific RNAi therapeutics context, as the balance and concentration of mutant versus wild-type transcripts feeding cellular translational machinery is disrupted. Notably, the principle of slicer-specific RNAi has been developed using overexpression systems and saturating concentrations of siRNAs, which are both far removed from physiologically relevant concentrations of RNA targets or indeed siRNA drugs. It is important at this point, however, to highlight that the understanding of target engagement kinetics in a RISC complex in isolation^52^ and in conjunction with its associated co-factors is too nascent to directly relate to the simplistic two-state model or even nearest-neighbor model of melting temperature (T_m_) calculation. Thus, while calculating a free-solution ΔT_m_ for the polymorphic targets might be simple, neither approach takes into account the allosteric effects of the RISC complex to target engagement, nor are the subcellular concentrations of key ionic solutes known or indeed stable. The net effect is that it is unclear at this point if a free-solution T_m_ value, whether computed or experimentally derived, effectively relates to the physiological conditions pertaining to RISC-substrate engagement and its preference to specific mismatches. Indeed, the position-specific tolerances to mismatched substrates demonstrated with miRNA and herein (e.g., position 5) point away from this precept.

On the other hand, a C→A transversion at position 11 resulted in only <1 log effect on novel 5′ ends starting at this position or position 12, identifying again a M→K substitution as detrimental (>3× log reduction onto elimination) to target recognition (Figure 6C). In stark contrast, any substitution at position 12 practically eliminated cleavages within the 3′ end of the hybridization site (Figure 6D). Crucially, as we report that cleavage beyond position 12 necessitates full complementarity across the central region of the guide strand, the requirement effectively locks in tandem the two CTCA runs in the siRNA22 guide strand hybridized to the target sequence. This would suggest that cleavages beyond the primary AGO2 active site are not on account of guide strand slippage, and they are a consequence of either AGO2 slippage around the guide strand-to-target duplex or a secondary enzymatically active site in AGO2. Alternatively, the lower 5′ RACE-SEQ coverage across positions 14–17, where the secondary CTCA run resides, may occlude slicing events among the replicon quasi-species. Attempting sequencing at this depth would be problematic, as this would compromise the ultra-low error rate of dual sequencing, whereas physical separation of the secondary cleavage products would be challenging due to the minimal size differences of the amplicons. Nonetheless, for the central CTCA run to slip upstream on the target and drive slicing, practically the entire 5′ end (5 of 7 bases) and 3′ end (4 of 9 bases) would need to be mismatched to the target (9 of 19 mismatches total). On balance, the bulk of evidence over mismatch tolerance to date,^3^ the observation of cleavage around positions 14–17 with siRNA19, which lacks any sequence repeats in this region, and the data of others implementing 5′ RACE-SEQ^19^ would argue against such a hypothesis and favor AGO2-driven secondary site cleavage as the most likely mechanism.

Notwithstanding such additional behaviors by AGO2, we propose that future studies on therapeutic siRNAs and indeed other endonucleotically active oligonucleotide therapeutics may find considerable value in pursuing RACE-SEQ analysis of partial or, preferably, full-length target transcripts incorporated within replicon systems. Capturing the detailed effects of specific base substitutions in such a semiquantitative manner may inform more precise off-target gene identification (e.g., by actively looking for target sequence-dependent, position-specific tolerances). We would also argue that inclusion of more than just the RISC hybridization site would be relevant in ensuring best possible maintenance of the structural sequence context, as suggested previously regarding microRNA recognition element reporter systems.^53^ Alternatively, exploring base-by-base variant susceptibility through RACE-SEQ on a replicon system may inform the extent to which known and unknown genomic polymorphisms in the population are susceptible to RNAi and antisense drugs. Thus, patient stratification and theranostic potential, an element of personalized cancer medicine increasingly required for new drug approval,^54^ would normally require the labor-intensive construction of reporter plasmid libraries encompassing the most common mutations among a patient population. This would rely on public domain information on polymorphism incidence, which is inherently biased by the limitations in population sampling encountered during database construction (e.g., dbSNP) and is compounded by researchers’ propensity to explore the effects of only the most common SNPs. In contrast, replicon construction is a relatively simple, single plasmid engineering process that substantially de-risks this bias, as it does not depend on patients, albeit with a tendency to prefer transversions to transitions. Thus, as we show here, all possible SNPs can be semiquantitatively analyzed for their susceptibility to a given RNA-acting drug through RNA-SEQ, in a minimally biased fashion, to pre-emptively inform clinical trial patient selection and, ultimately, drug prescription, thereby maximizing the chances of achieving a therapeutic effect. Conversely, the method may offer value in monitoring the mutational escape propensity of tumors, quantifying in a mechanism-of-action-dependent way the impact of specific mutations within a target sequence.

In summary, by implementing advances in SEQ data handling, we present a relatively simple, open-access pipeline for RACE-SEQ data processing. Moreover, we advance the approach to using replicon systems for exploring in detail how target mismatches to guide strands affect RNAi-mediated target cleavage. Furthermore, our data offer additional insights on the roles of seed and primary cleavage point base pairing for both canonical Slicer activity at position 10–11 as well as what appears to be secondary Slicer activity (Slicer slippage) closer to the 3′ end of the guide strand. By using a natively error-prone RNA replicon system, we were also able to scrutinize base by base how substitutions in a target affect its recognition by RISC. This approach may significantly empower a priori drug susceptibility research for emerging RNA-targeting therapeutics. Thus, using this patient-free, bench-based, and minimally biased method, semiquantitative data can be produced to inform patient selection in clinical trials. Moreover, we show how mismatches, even within the primary Slicer active site, can still give rise to novel 5′ ends toward the 3′ end of the guide strand and perhaps even within the seed region. Whether these actions are driven by AGO2 or emerging co-factors such as GIGYF2^55^ remains to be determined.

## Materials and Methods

### Datasets

We revisited all three RACE-SEQ datasets originally reported by Denise et al.^18^ Each of these datasets corresponds to the Slicer products generated on the HCV Con1B genome pFK I389 Lucubineo EI NS3-3′ ET (GenBank: AJ238799) by synthetic analogs of the three primary siRNAs expressed by TT-034, siRNA6, siRNA19, and siRNA22. The target region of siRNA6 (5′-CGAAAGGCCTTGTGGTACTG-3′) is located in the 5′ UTR on position 272–291, whereas the target regions of siRNA19 (5′-GTCAATTCCTGGCTAGGCAACAT-3′) and siRNA22 (5′-CAAGCTCAAACTCACTCCAAT-3′) are located in the NS5B gene, which codes for the viral RNA-dependent RNA polymerase, on positions 9,095–9,117 and 9,478–9,498, respectively. Briefly, we used Illumina reads supplied as individual .fastq files after sequencing adaptor and quality-control trimming. In a 5′→3′ orientation, the reads consisted of the 5′ RACE adaptor (30 bp; 5′-GGACACUGACAUGGACUGAAGGAGUAGAAA-3′) ligated at the novel 5′ ends generated on the HCV genome by Slicer action resulting into an ∼80-bp read length.

### Operating System and Software Environments

All the bioinformatics analyses for this project were undertaken on a Bio-Linux 8 workstation within an Ubuntu Linux 14.04 LTS base^56^ and bioinformatics packages updated to their latest available versions using the default update advisor. Additional packages, such as Cutadapt v1.11, FASTX-Toolkit v0.0.14, and BEDTools v2.26.0, were downloaded manually from GitHub and their respective online repositories. Furthermore, the R libraries “Biostrings,” “tools,” “ShortRead,” and “stringi” were downloaded and installed from the Rstudio v0.99.903 online repositories (Comprehensive R Archive Network [CRAN]) and were utilized for .fasta sequence and character string manipulation.^57^

### RACE Adaptor Trimming

Denise et al.^18^ removed RACE adapters semi-manually by constructing a custom R script (v2.10.1) using the Bioconductor “ShortRead” library.^58^ We used Cutadapt^59^ instead, to enable simple and tuneable data processing. Thus, the following parameters were invoked: *-g* to set adaptor location on the 5′ end of the reads, *-e* to set mismatch error tolerance for adaptor alignment, *--no-indels* to set insertion and deletion tolerance to 0 during adaptor alignment, *--discard-untrimmed* to discards reads lacking the full adaptor sequence, and *-m* to discards sequences shorter than a given number of nucleotides before and after adaptor trimming. After adaptor removal, quality analysis was performed by FastQC (v0.11.3) to check per-base/sequence quality scores, sequence length distribution, and adaptor content/contamination. To reduce computational overheads, we implemented fastx_collapser from the FASTX-Toolkit, which reduces the dataset size and identifies all the unique sequence products.

### Read Alignment

As per Denise et al.,^18^ we used the ultra-fast, memory-efficient Burrows-Wheeler transformation algorithm Bowtie v1.1.1^60^ to align reads to the Con1B reference sequence. Index files of the Con1B reference genome were constructed using the “*bowtie-build*” command. The Bowtie command was then invoked with the following specific parameters: *-p* that sets the number of threads that will run in separate cores/processes in parallel, *-S* that sets the output in .sam format, *-k* that outputs only the given number of valid alignments per read, *-v* that sets the number of mismatches that are allowed during alignment, and *--al-gz* and --*un-gz* that output two separate files containing the aligned and unaligned reads, respectively, in .gz compression for further analysis and processing. Given the prevalence of Illumina and Ion Torrent systems worldwide, beyond Bowtie1 and 2, we additionally evaluated the impact of Torrent Mapping Alignment Program (TMAP), the aligner preferred for Ion Torrent systems, and we observed no appreciable differences in novel 5′ end detection, provided all other analytical parameters (e.g., >Q30 trimming) are maintained.

### 5′ End Counting

The Denise et al.^18^ pipeline imported the .sam files into R and utilized the “Rsamtools” library command “pileup” to count the 5′ end of the reads. Here, the Bowtie .sam output file was viewed using SAMtools v0.1.19 invoking the “*view*” command from the command line.^61^ The parameters *-bS* were then used to transcode files into the compressed and indexed .bam binary format, a machine-readable but smaller sized file. To count the 5′ ends of the reads, the “*genomCoverageBed*” command (BEDtools v2.26.0)^62^ was invoked. Specific parameters included the following: *-ibam* that reads the input files in .bam format, *-d* that reports the depth at each genomic position of the reference, and *-5* that processes and calculates the coverage of the 5′ positions only instead of the entire read interval. The output of this command is a 3-column .txt file that contains the name of the reference sequence, the nucleotide position, and the depth of coverage in every position.

### Graph Plotting

To plot the graphs of the read counts, we used the R (v3.3.1) statistical language and the Rstudio v0.99.902 graphical interface.^63^ Initially, the “*biostrings*” and “*tools*” libraries were loaded in an Rstudio session. The reference sequence was then imported and manipulated as a string to attach it to the .txt table containing the read counts. The percentage and log_10_ transformation of the read counts were then calculated and attached on the table, which was exported as a comma-separated value (CSV) object. User-imported settings were then used to point to the region of interest and the desired nucleotide positions to be plotted. Finally, a PDF file was created where percentage and log_10_ bar plots were generated and attached. The bar plots were generated with the R base bar plot command from the corresponding linear and log_10_ values. Legends, names, and axis values were then imported using the text command. An example of the resulting graph is reported in Figure S5. The resulting “RACE-SEQ lite” pipeline is an open-source data handling tool available in the GitHub repository (https://github.com/pantastheo/RACE-SEQ-lite).

### Investigating Mismatch Tolerance

To examine how RNAi cleavage is affected by mismatches on the target sequence, we manipulated mismatch tolerance parameters during read alignment with Bowtie. Both Bowtie1 and Bowtie2 were evaluated within this analytical process. Using Bowtie1, we explored the effect of increasing the number of random mismatches between the RACE-SEQ reads and the reference genome. This was achieved by carrying out alignments iteratively, each time modifying the *-v* mismatch parameter from 0 to 1, 2, or 3 and plotting the resulting data. Confusingly for RNAi users, however, Bowtie 2 uses the term “seed” to define and extract substrings of deep sequencing reads during so-called “multiseed heuristic” alignment.^64^ Although the net result is comparable to Bowtie1, Bowtie1 tries to align the entire read in an end-to-end fashion, which is inherently a slower process than substring processing.^60^ However, substring processing impacts on 5′ end definition for RACE-SEQ by nature of eliminating the key 5′ bases from substrings. Thus, we set the following: *-n* to set the number of mismatches permitted in a substring of the sequence and *-l* to set the length of the bps constituting the substring sequence. Setting the error tolerance explicitly for the seed length specified and the length of the substring to be used, we automated the whole process for one and zero mismatches and three different seed lengths as follows: “*-n0 -l3*,” “*-n0 -l5*,” “*-n0 -l10*,” “*-n1 -l3*,” “*-n1 -l5*,” and “*-n1 -l10.*” We then ran the dataset through our pipeline and looked for novel 5′ ends flanking the expected cut site (Figure S6).

## Author Contributions

Conceptualization, S.A.M; Methodology, S.A.M.; Software, P.I.T. and C.K.K.; Validation, P.I.T. and L.U.; Formal analysis, P.I.T., E.S., and S.A.M.; Investigation, P.I.T. and C.K.K.; Data Curation, P.I.T., L.U., and S.A.M.; Writing – Original Draft, P.I.T. and S.A.M.; Writing – Review and Editing, L.U. and S.A.M.; Visualization, P.I.T.; Supervision, S.A.M.; Funding Acquisition, S.A.M.

## Conflicts of Interest

The authors declare no known conflicts of interest.

## Supplemental Information

Document S1. Figures S1–S7 and Table S1

Document S2. Article plus Supplemental Information
