# Supplementary material for: Profiling the Mismatch Tolerance of Argonaute 2 through Deep Sequencing of Sliced Polymorphic Viral RNAs
Source: Mol Ther Nucleic Acids. 2017 Aug 24;9:22–33. doi: 10.1016/j.omtn.2017.08.010 (PMC5602524; doi:10.1016/j.omtn.2017.08.010)
Supplement: Document S1. Figures S1–S7 and Table S1 [file mmc1.pdf]

OMTN, Volume *9*

Supplemental Information

Profiling the Mismatch Tolerance of Argonaute 2 through Deep Sequencing of Sliced Polymorphic Viral RNAs

Pantazis I. Theotokis, Louise Usher, Christopher K. Kortschak, Ed Schwalbe, and Sterghios A. Moschos

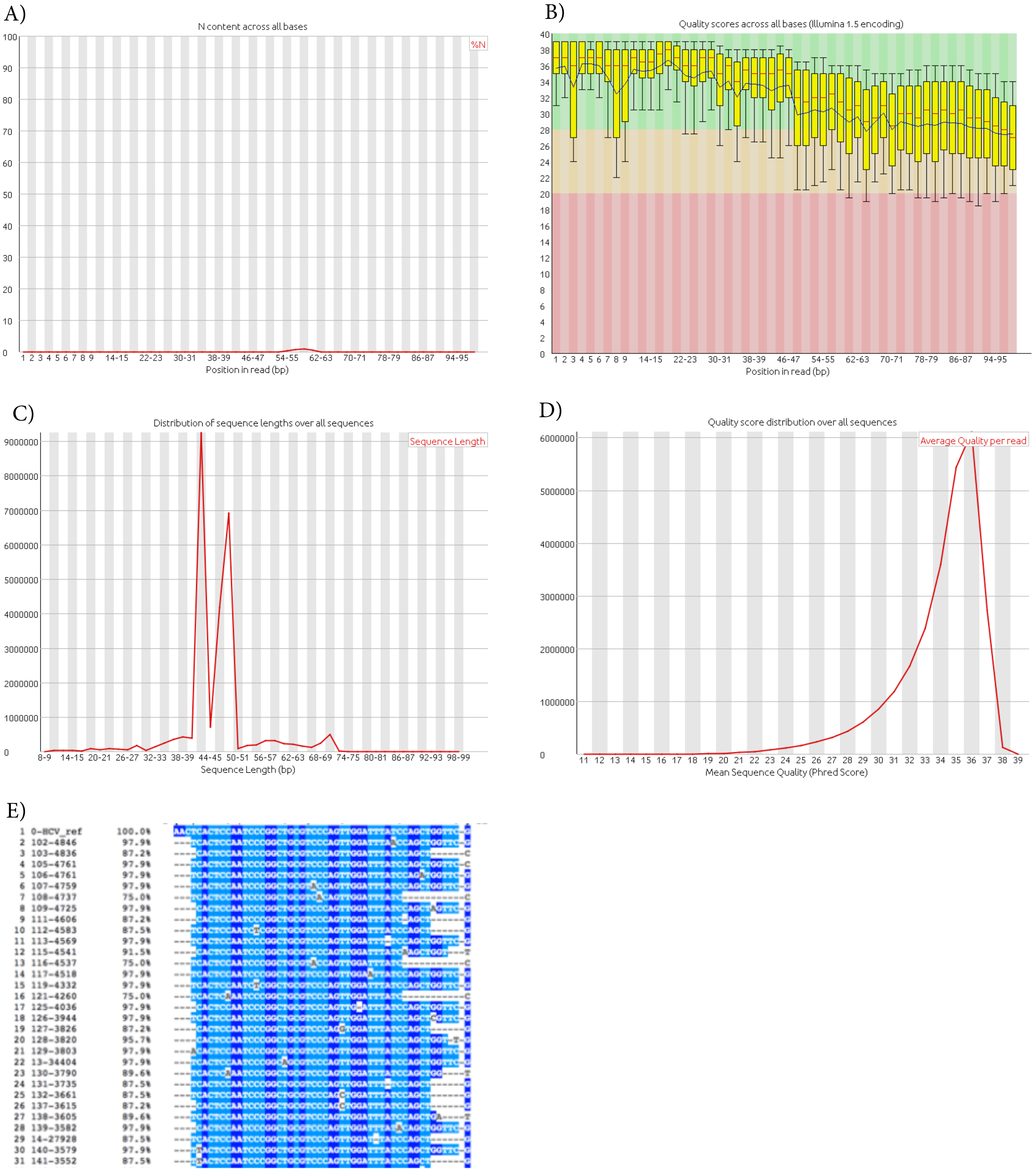

Supplemental Figure 1. FastQC Quality Control report output excerpt after 5' RACE adapter trimming using Cutadapt and visualisation of the nature of unaligned reads. A) Limited incidence of unresolved bases (N content) across the RACE-SEQ reads. B) Per base PHRED Quality score distribution does not fall under Q20 in 5' RACE adapter-treamed reads. C) Trimmed read size distribution profile. D) Read PHREAD score distribution across all reads after RNA adapter trimming. E) Visualising in MView a T-Coffee multiple sequence alignment of collapsed 5' RACE SEQ reads indicates that a large fraction of reads might be arising from HCV quasi-species featuring insertions, deletions and mutations (Purines in dark blue, pyrimidines in light blue)

Supplemental Table 1. Extended Cutadapt summary report following RACE adapter removal.

```
biolinux@E-Laptop-38[NEW_old] cutadapt -gGGACACTGACATGGACTGAAGGAGTAGAAA -e0 --no-
indels -m10 --discard-untrimmed -o trimmed_009.fastq.gz siRNA_009.fastq.gz
This is cutadapt 1.10 with Python 2.7.6
Command line parameters: -gGGACACTGACATGGACTGAAGGAGTAGAAA -e0 --no-indels -m10 --
discard-untrimmed -o trimmed_009.fastq.gz siRNA_009.fastq.gz
Trimming 1 adapter with at most 0.0% errors in single-end mode ...
Finished in 1351.46 s (22 us/read; 2.77 M reads/minute).

=== Summary ===

Total reads processed:              62,334,988
Reads with adapters:                26,412,283 (42.4%)
Reads that were too short:             609,109 (1.0%)
Reads written (passing filters):    26,238,713 (42.1%)

Total basepairs processed: 4,598,424,045 bp
Total written (filtered):  1,203,983,899 bp (26.2%)

=== Adapter 1 ===

Sequence: GGACACTGACATGGACTGAAGGAGTAGAAA; Type: regular 5'; Length: 30; Trimmed:
26412283 times.

No. of allowed errors:
0-30 bp: 0

Overview of removed sequences
length     count expect     max.err    error counts
3    18777 973984.2   0    18777
4    7404  243496.0   0    7404
5    2050  60874.0    0    2050
6    593   15218.5    0    593
7    380   3804.6     0    380
8    290   951.2 0    290
9    372   237.8 0    372
10   603   59.4  0    603
11   453   14.9  0    453
12   640   3.7   0    640
13   124   0.9   0    124
14   307   0.2   0    307
15   1484  0.1   0    1484
16   1131  0.0   0    1131
17   1419  0.0   0    1419
18   653   0.0   0    653
19   3507  0.0   0    3507
20   1922  0.0   0    1922
21   7440  0.0   0    7440
22   5714  0.0   0    5714
23   12998 0.0   0    12998
24   50732 0.0   0    50732
```

Continued in the next page

25	117156	0.0	0	117156
26	155802	0.0	0	155802
27	289944	0.0	0	289944
28	264827	0.0	0	264827
29	419351	0.0	0	419351
30	24098522	0.0	0	24098522
31	877221	0.0	0	877221
32	58377	0.0	0	58377
33	3779	0.0	0	3779
34	468	0.0	0	468
35	265	0.0	0	265
36	212	0.0	0	212
37	332	0.0	0	332
38	253	0.0	0	253
39	489	0.0	0	489
40	332	0.0	0	332
41	334	0.0	0	334
42	805	0.0	0	805
43	260	0.0	0	260
44	386	0.0	0	386
45	634	0.0	0	634
46	355	0.0	0	355
47	534	0.0	0	534
48	334	0.0	0	334
49	526	0.0	0	526
50	335	0.0	0	335
51	133	0.0	0	133
52	149	0.0	0	149
53	249	0.0	0	249
54	118	0.0	0	118
55	654	0.0	0	654
56	103	0.0	0	103
57	11	0.0	0	11
58	5	0.0	0	5
59	3	0.0	0	3
60	2	0.0	0	2
62	2	0.0	0	2
64	11	0.0	0	11
65	1	0.0	0	1
66	7	0.0	0	7
67	1	0.0	0	1
68	3	0.0	0	3
72	1	0.0	0	1
73	1	0.0	0	1
80	1	0.0	0	1
82	1	0.0	0	1
95	1	0.0	0	1

biolinux@E-Laptop-38[NEW_old] [2:47AM]

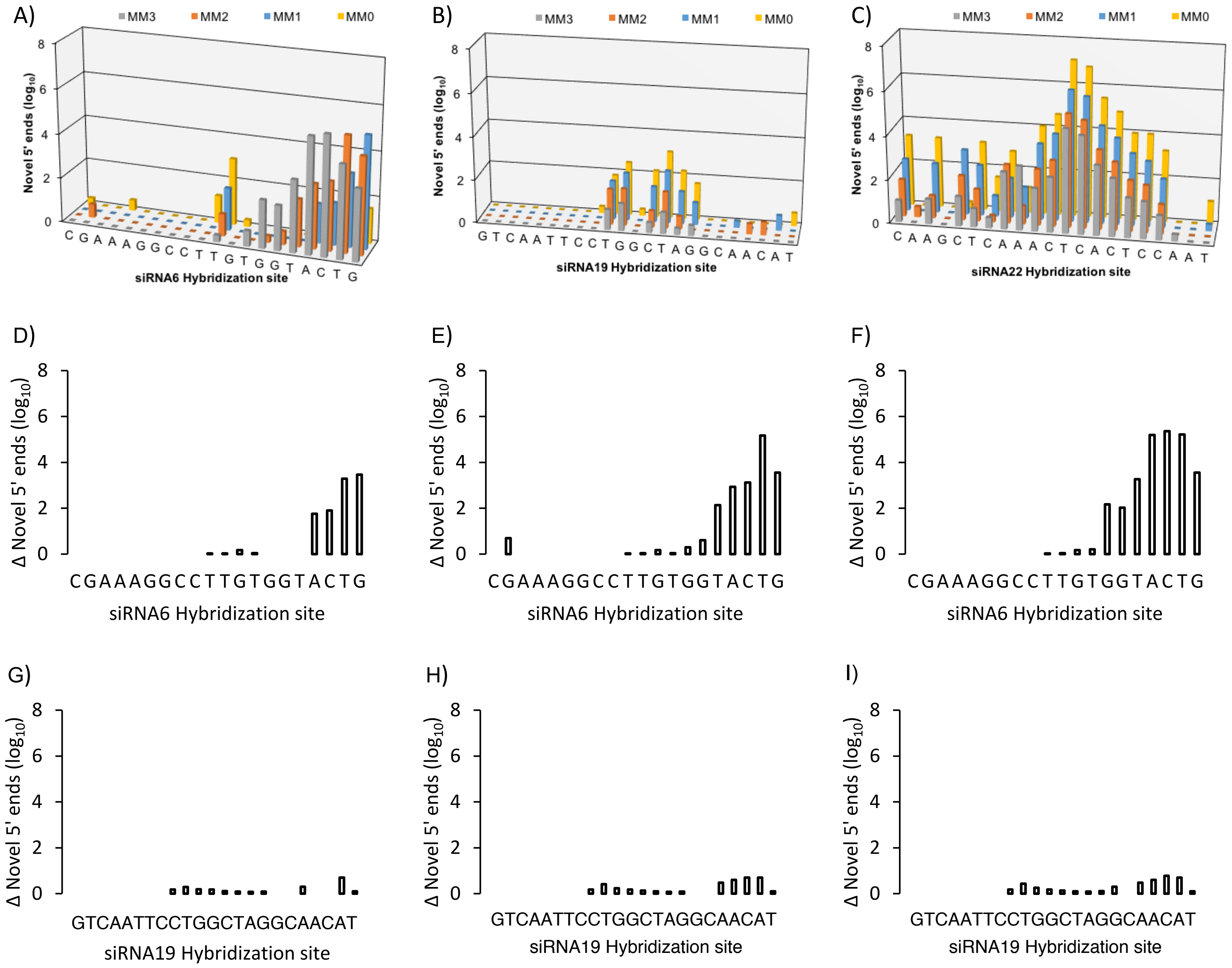

Supplemental Figure 2: Changes in 5'-RACE-SEQ product profiles at increasing levels of random mismatch tolerance during data alignment to the corresponding target sequence. The incremental increases in novel 5' end counts when accepting progressively more (MM0-MM3) mismatches are plotted for siRNA6 (A), siRNA19 (B) and siRNA22 (C), synthetic siRNA analogues to the anti-HCV shRNA encoded in TT-034.The log-scale changes in novel 5' profiles generated under 1 (D, G), 2 (E, H) or 3 (F, I) maximum random mismatches are displayed for the synthetic analogs of the anti-HCV-encoded TT-034 siRNA6 (A-C) and siRNA19 (D-F).

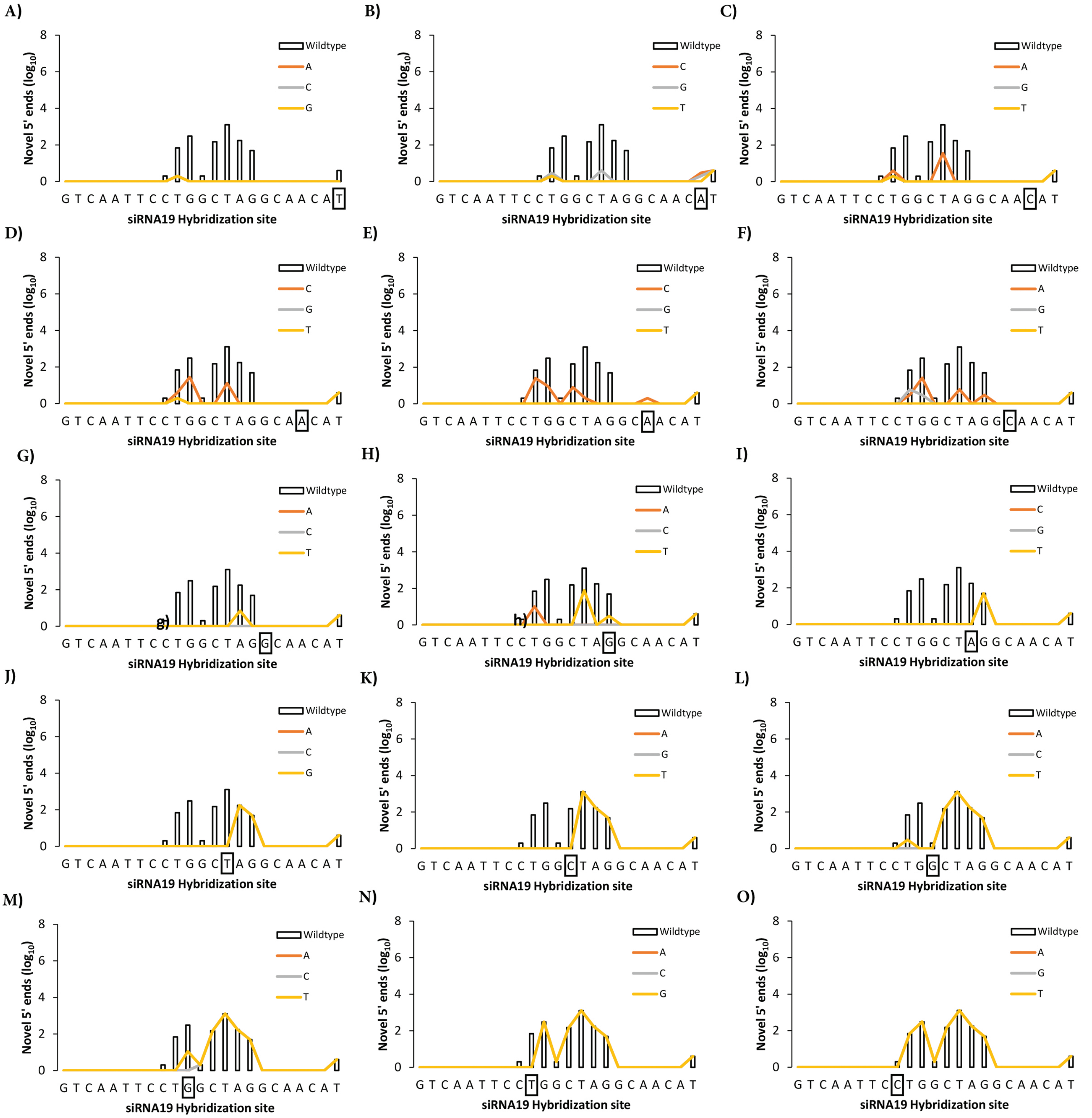

Supplemental Figure 3. Effect of specific nucleotide substitutions on RACE-SEQ profiles across the 15 Watson-Crick pairing positions from the 5' end of the siRNA 19 guide strand directed against the Con1B HCV replicon. A boxed nucleotide on the X axis highlights the base substituted within each panel. A differentially coloured line represents the effect of each possible base substitution within the boxed base.

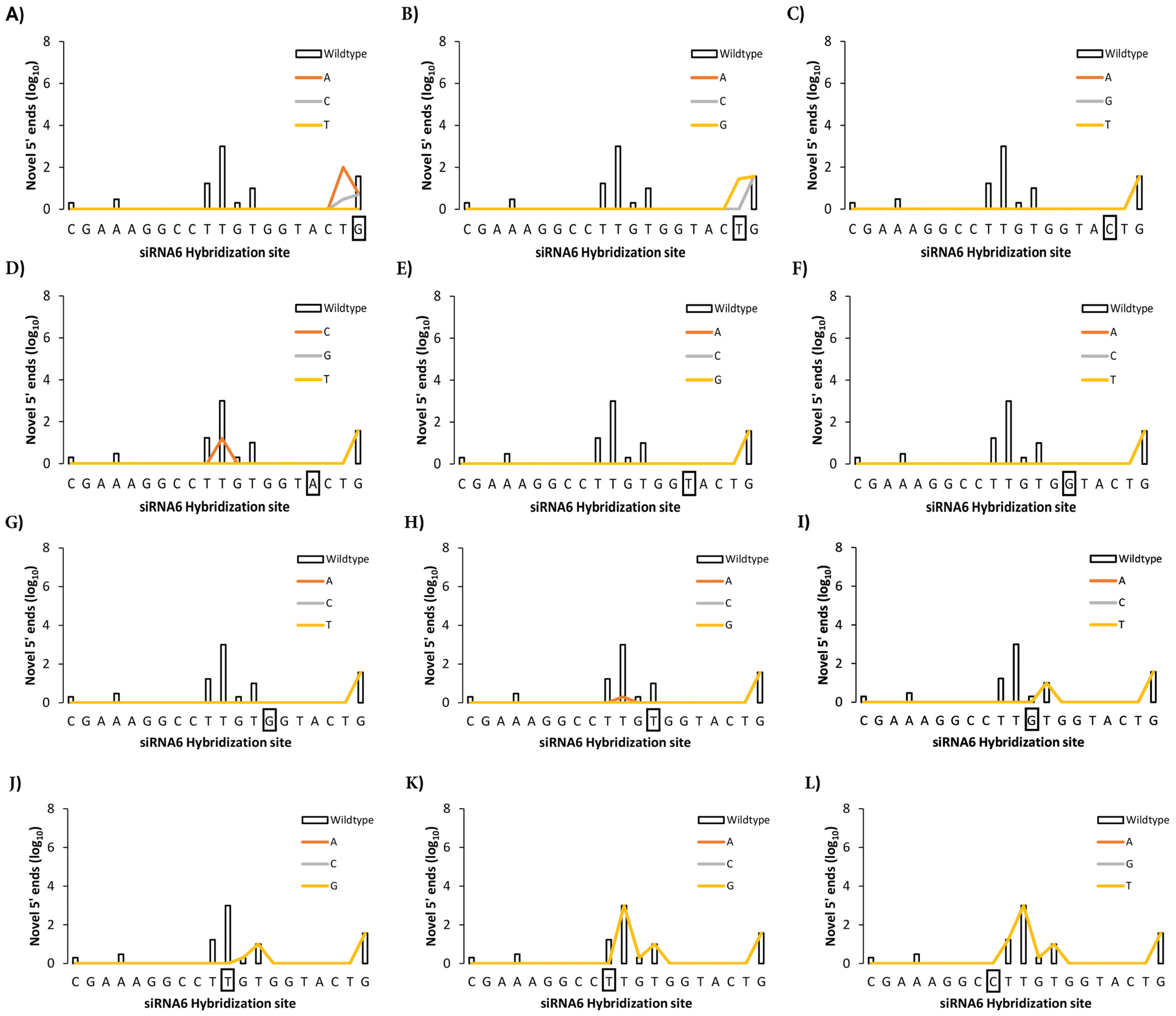

Supplemental Figure 4. Effect of specific nucleotide substitutions on RACE-SEQ profiles across the 12 Watson-Crick pairing positions from the 5' end of the siRNA 6 guide strand directed against the Con1B HCV replicon. A boxed nucleotide on the X axis highlights the base substituted within each panel. A differentially coloured line represents the effect of each possible base substitution within the boxed base.

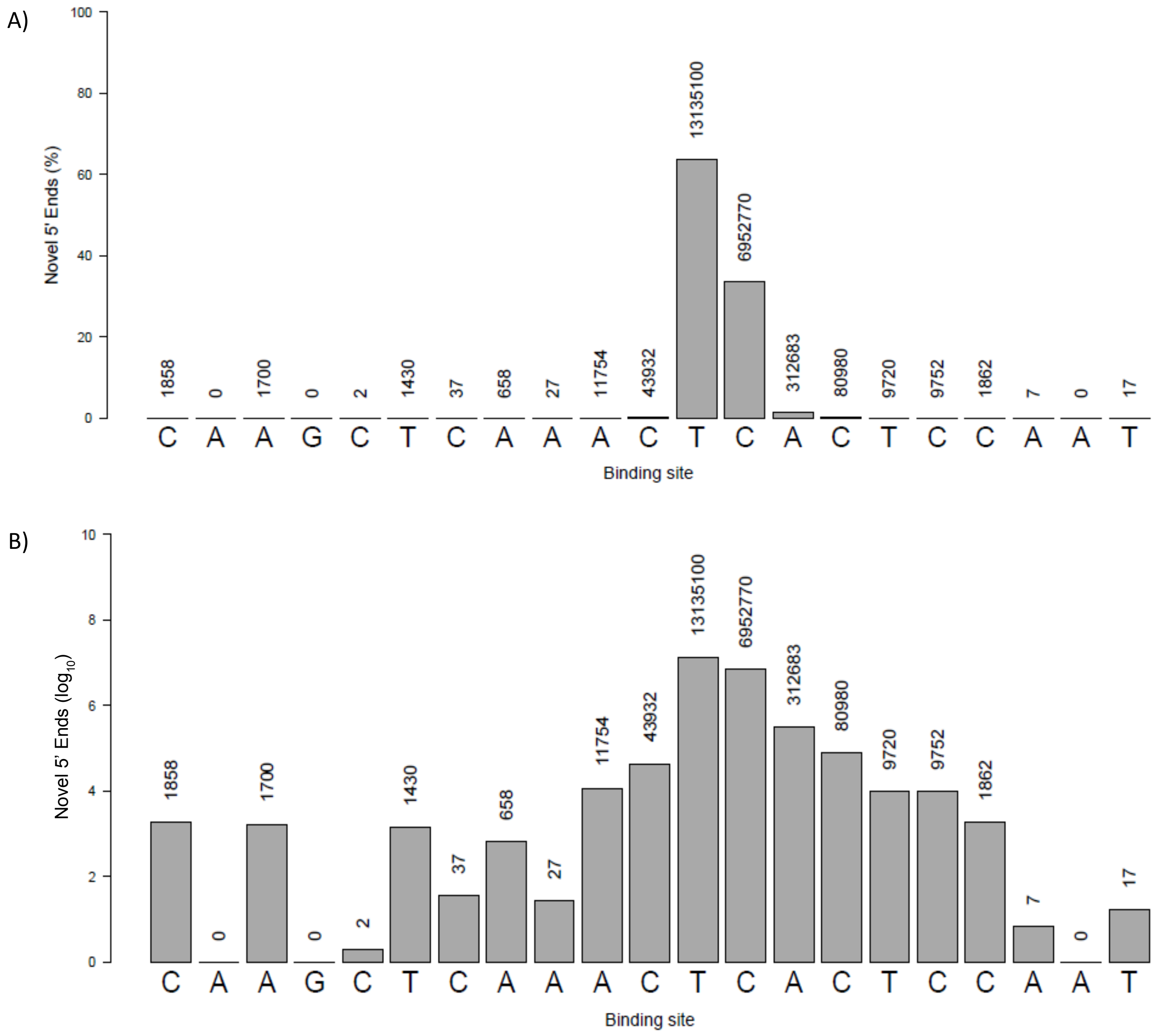

Supplemental Figure 5. Sample output of the RACE-SEQ lite pipeline processing 5'-RACE SEQ data.

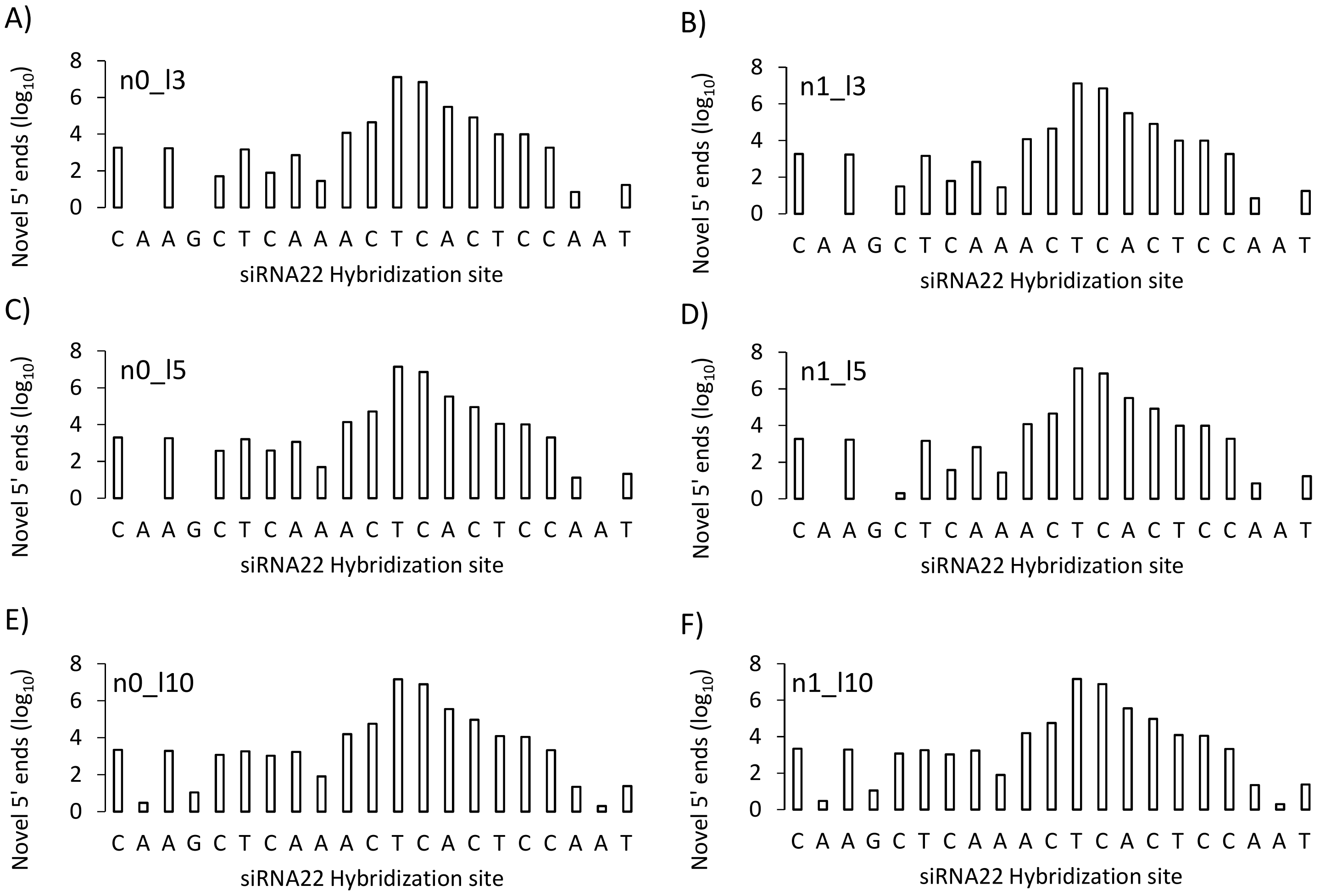

Supplemental Figure 6. Impact of Bowtie2 seed substring length parameter adjustment on RACE-SEQ data outputs highlights the incompatibility of this aligner with RACE-SEQ data processing. As Bowtie2 uses substrings of a read to map faster reads onto a reference genome, we explored the effect of substringth length of 3 (A, B), 5 (C, D) and 10 (E, F) nucleotides with no (A, C, E) or one (B, D, F) mismatch permitted during alignment for the RACE-SEQ products of the TT-034-encoded siRNA22. The parameter specification is represented in each panel with -n identifying the number of mismatches and -l the seed length.

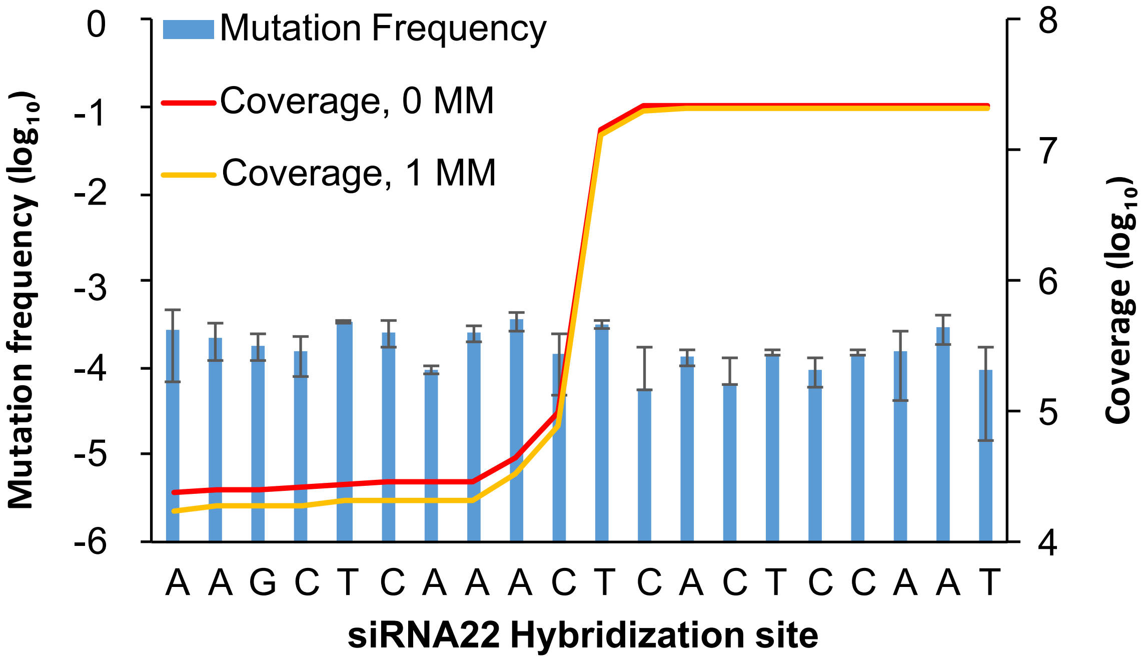

Supplemental Figure 7: Per base coverage in replicon systems defines RACE-SEQ sensitivity to quasi-species susceptibility to slicer cleavage. The per-base mutation frequency of HCV replicons (primary Y axis) across the siRNA22 target site (X axis) was reported as 1:1,000 to 1:10,000 by Geller *et al*,[44] setting a 4 log per base coverage threshold for effective detection of HCV quasi-species. Whilst coverage of the siRNA22 hybridization site exceeds this threshold, the 5' sensitivity of 5' RACE-SEQ results in greater coverage (secondary Y axis) downstream from the primary slicer cleavage point (scissors). Coverage is also not substantially increased by permitting 1 mismatch (MM) in 5' RACE-SEQ read alignment to the reference genome; together with the $8.4x10^{-8}$ per base error rate in duplex sequencing used to generate this data, these mismatches are likely the

outcome of quasi species sequencing and not PCR/SEQ error propagation.
